# Family connections vs treatment at usual optimized in the treatment of relatives of people with suicidal behavior disorder: study protocol of a randomized control trial

**DOI:** 10.1186/s12888-022-03965-5

**Published:** 2022-05-15

**Authors:** José H. Marco, Sara Fonseca, Isabel Fernandez-Felipe, Azucena García-Palacios, Rosa Baños, Sandra Perez, Joaquín Garcia-Alandete, Verónica Guillen

**Affiliations:** 1 Departamento de Personalidad, Evaluación y Tratamiento Psicológico, Universidad de Valencia, Facultad de Psicología, España. Avda. Blasco Ibañez, 21, Valencia, España; 2 Ciber Fisiopatologia Obesidad y Nutrición (CB06/03), Instituto Salud Carlos III, Madrid, España; 3 Universitat Jaume I de Castelló, España. Facultad de Ciencias de la Salud, Avda Sos Baynat, S/N, Castellón, España

**Keywords:** Family Connections, Suicidal Behavior Disorder, Suicide Attempts, Caregivers, Relatives, Dialectical Behavioral Therapy

## Abstract

**Background:**

Relatives of people diagnosed with suicidal behavior disorder (SBD) feel guilty, afraid, hopeless, depression and anxiety. It is necessary to help the relatives of people with SBD to reduce their discomfort and burden. Family Connections (FC) is a program that has been shown to be effective in reducing burden, depression, and anxiety, and increasing dominance and validating behaviors in relatives of people with borderline personality disorder. However, there are no RCTs that demonstrate the efficacy of the FC program in patients with SBD. Our research team adapted FC for relatives of people with SBD for delivery in the Spanish population (FC-SBD). The FC-SBD program contains 12 two-hour sessions held once a week. The first aim is to verify the efficacy of the FC-SBD intervention for relatives of people diagnosed with SBD in a randomized control trial with a Spanish sample. The second objective is to analyze the feasibility and acceptance of FC-SBD in relatives. The third aim is to analyze whether the changes produced in the psychological variables in the relatives after the intervention are related to changes in the psychological variables of the patients. This paper presents the study protocol.

**Methods:**

The study design consists of a two-arm randomized controlled trial with two conditions: FC-SBD or Treatment as usual optimized (TAU-O). Participants will be relatives of patients who meet DSM-5 criteria for SBD. The caregivers` primary outcome measures will be the BAS. Secondary outcomes will be DASS-21, FES, DERS, QoL. The patient’s primary outcome measures will be the frequency of critical incidents with the family member with SBD. Secondary measures will be the INQ, PHQ-9, OASIS. Participants will be assessed at pretreatment, post-treatment, and 6-month follow-up. The intention-to-treat principle will be used when analyzing the data.

**Discussion:**

This study will provide results that confirm the efficacy of the FC-SBD in relatives of people with SBD. These results will also confirm its good acceptance by family members and help us to find out whether it is a good program to improve the prevention of suicidal behaviors in the family environment.

**Trial registration:**

ClinicalTrials.gov ID: NCT05157607. Registered 15 December 2021.

## Background

Suicide is a major public health problem [1]. However, in the past 45 years suicide rates have increased by 60% globally, and suicidal behavior is currently one of the leading causes of death worldwide [2], affecting more than 800,000 people each year [3]. Although suicide prevention is difficult, there is some consensus that prior suicide attempts are the main indicator of completed suicide [4]. Other important risk factors are suicidal ideation, the frequency and number of different methods of self-harm, hopelessness, borderline personality disorder, post-traumatic stress disorder, impulsivity, depression [5], being unemployed, living alone, having low social support, or schizophrenia [6].

Meta-analytic studies indicate that two types of interventions have been shown to be effective in reducing suicide attempts. On the one hand, Dialectical Behavior Therapy (DBT), randomized controlled studies with a two-year follow-up found that DBT was superior to the usual therapy performed by psychologists and psychiatrists who are experts in the treatment of suicide. Thus, DBT reduced suicide attempts by 50% compared to expert suicide therapy [7]. DBT was also more effective in reducing emergencies, doctor visits, and hospital psychiatric care for suicide. Other studies have found that DBT for people with borderline personality disorder (BPD) produced a reduction in the frequency and severity of suicide attempts and non-suicidal self-harm one year after receiving DBT [8]. On the other hand, Cognitive Behavioral Therapy (CBT) [9] has been shown to be effective in reducing the risk of suicide, suicidal ideation, and suicidal behavior in adult patients and in individual [10] and group therapy, reducing the probability of suicide attempts by 50% compared to the usual treatment [11]. However, it is striking that excellent review studies carried out in the adult population [12, 13] do not take into account whether a specific intervention was carried out with families or caregivers and whether they influenced the effectiveness of the treatment.

Suicide attempts are a critical challenge faced by families living with a patient. Family members feel guilty, afraid, and hopeless about the possibility of suicide [14], and this increases the risk of depression and anxiety [15, 16]. Furthermore, it is difficult for family members to recognize suicide attempts, which makes prevention difficult [17]. Family members of people who have attempted suicide are at a high risk of having medical and psychological problems, and, more importantly, they have a higher risk of committing suicide [18]. They tend to report that they are not cared for adequately and that they need help and want to know and understand the treatments the patients receive [14], as well as the skills they may need to relate to them [19].

All of these studies suggest that the needs of family members of people with suicide attempts are not adequately addressed in the current treatments for people with suicide attempts. Therefore, it is necessary to help the relatives of people with suicidal behaviors to reduce the discomfort and burden they experience by giving them information and skills to improve their relationship with patients. In this way, suicide attempts could be prevented / reduced in the family context.

Treatment programs designed specifically for relatives of patients with suicide attempts are scarce. However, action models have been designed to meet the needs of families of people with mental illnesses such as schizophrenia, bipolar disorder, and depression [20], or for relatives of people with eating disorders [21]. In the case of BPD (patients who are at a high risk of suicide attempts), psychoeducation groups can be held for families in order to provide information about the disease and help them understand some of the behaviors of their sick relatives, thus improving the relationship and family coexistence [22].

“Family connections (FC): a program for relatives of persons with BPD” [23] is an adaptation of DBT skills training [24] designed for use with relatives of patients with BPD by professionals or family members who have previously completed a training course in order to lead other groups of family members. The FC program contains 12 two-hour sessions held once a week. The content of the intervention program is divided into six modules and includes psychoeducation about BPD and how it affects family functioning, skills adapted from the DBT program (individual, family, and relational skills, validation exercises, and problem-solving skills), and peer support. All the modules include specific practical exercises and homework assignments. In addition, throughout the FC program, there is a forum where participants can build a support network. Previous non-controlled clinical trials [23, 25–29] found that, after the FC intervention, significant decreases were observed in the subjective experience of burden, perceived discomfort, depression, and grief, and the relatives’ coping strategies improved. These changes were maintained at the three-month follow-up.

Furthermore, in a previous study, Rajalin [17] adapted the FC program in an open trial with relatives of people who had attempted suicide. The relatives had no clinical diagnosis, and the patients were being treated in a suicide prevention program. The results indicated that, after treatment, the general well-being of the family members increased, the feeling of burden and level of discomfort and anxiety decreased, and the relationship with the patient with suicide attempts improved. In addition, the criticism expressed and the expression of emotions decreased. Therefore, the results of this pilot study suggest that the FC program tailored to relatives of patients with suicide attempts may be effective in improving well-being and reducing the burden of illness in relatives. The main limitation of this study is that it is a pilot study with no control group or randomization of the participants.

Therefore, in order to advance in this line of work and improve the clinical situation and quality of life of relatives of patients with suicidal behaviors, it is necessary to have evidence-based intervention protocols focused on the relatives, and to do so it is necessary to submit them to tests of efficacy and efficiency in controlled studies.

This study has several aims. The first objective is to verify the efficacy of the FC intervention for relatives of people diagnosed with Suicidal Behavior disorders (SBD) in an RCT with a Spanish sample of participants from mental health services. The second objective is to analyze the feasibility and acceptance of FC-SBD in relatives of people with SBD. The third objective is to analyze whether the changes produced in the psychological variables in the relatives after the intervention are related to changes in the psychological variables of the patients. The fourth objective is to analyze whether improvements in emotional regulation, validation, control and empowerment, and family functioning predict reductions in burden, anxiety, and depression, and improvements in the quality of life of relatives. Fifth, we will analyze the opinions, preferences, and perceptions of the relatives in both treatment conditions.

We propose the following hypotheses: a) After the intervention, all the participants in the two treatment conditions, Family Connections (FC-SBD) or Treatment as usual optimized (TAU-O), will improve their level of burden, anxiety, depression, quality of life, validation, dominance, and empowerment, and critical incidents in the family environment will be reduced (self-harm, suicide attempts, arguments, etc.). However, the participants in the FC-SBD condition will improve more than those in the TAU-O condition; b) After the intervention, both interventions will have good acceptance by the participants, but the FC-SBD condition will have greater acceptance; c) The changes produced in the psychological variables in the relatives after the intervention will be related to changes in the psychological variables of the patients; and d) The improvement that may occur in the family members with regard to burden, anxiety, depression, and quality of life will be predicted by the improvements in emotional deregulation, validation skills, empowerment, and family functioning.

In this article, we present the study protocol.

### Methods and design

#### Study design

**Fig. 1:**
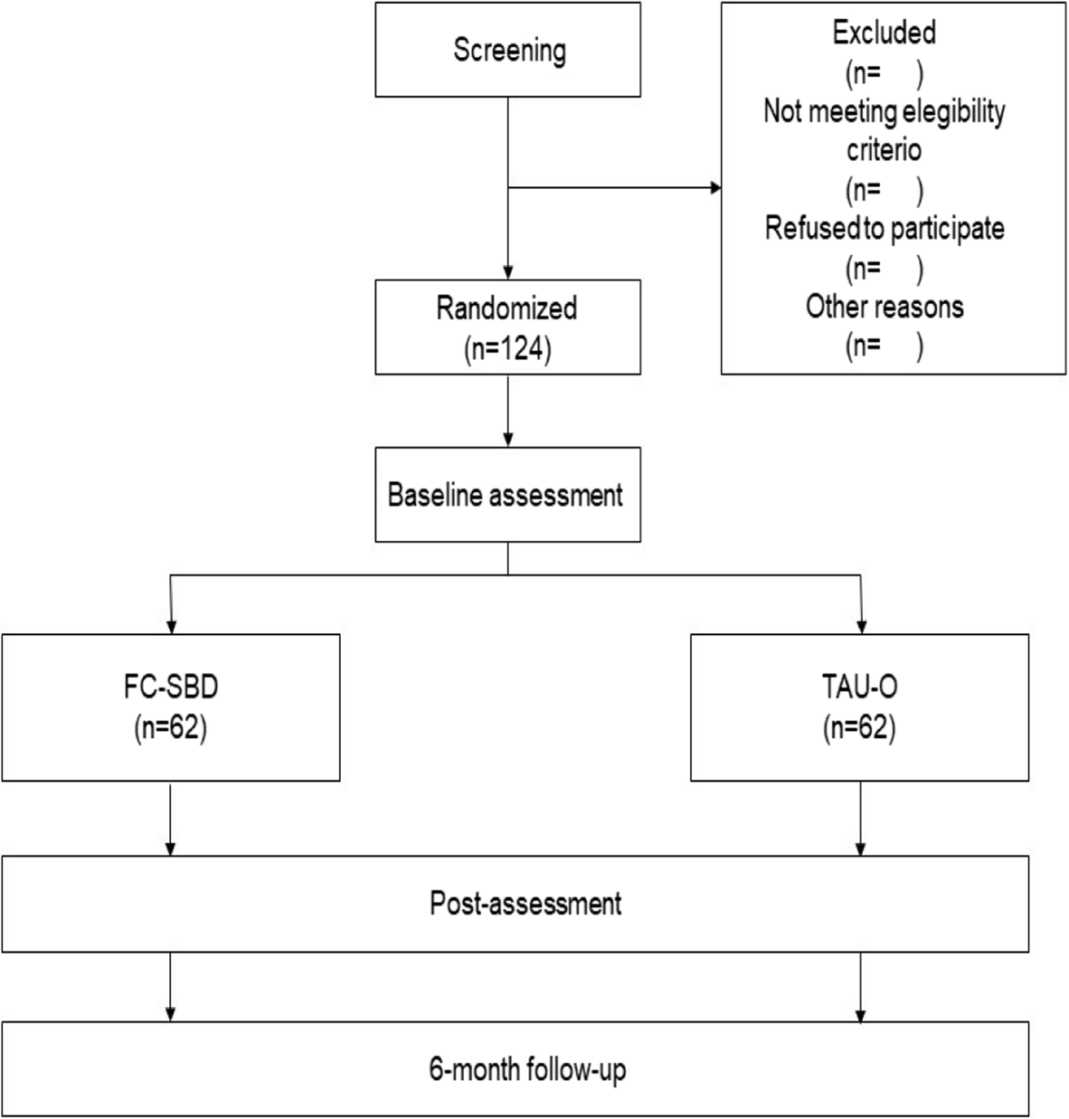
Flow chart of study. Note: FC-SBD= Family Connections for Suicidal Behavior Disorder; TAU-O = Treatment at Usual Optimized

This study is a superiority trial. The study design consists of a two-arm randomized controlled trial (RCT). On the one hand, there will be two conditions: Family Connections (FC-SBD) or Treatment as usual optimized (TAU-O), and family members will be randomized to one of the two groups. Family members will be randomized taking into account that if a patient has more than one family member attending the group, they will be randomized together to be included in the same condition. The effects of the treatment will be measured before starting the group, afterwards, and at the six-month follow-up in order to know whether the effects are maintained in the long term. Figure 1 shows the flow chart. This protocol will follow the CONSORT statement (Consolidated Standards of Reporting Trials, http://www.consort-statement.org) [30, 31] and the SPIRIT guidelines (Standard Protocol Items: Recommendations for Interventional Trials) [32, 33].

### Sample size

The sample size for this study was generated from effect sizes found in other studies with relatives of people with mental disorders. In the literature, we found a controlled study by Grenyer et al. [22] on a psychoeducational group intervention protocol for family members of people with borderline personality disorder. On the one hand, they found medium to large effect sizes for dyadic adjustment (d=0.78) and family empowerment (d=1.4); on the other hand, they found medium effect sizes for burden (d=0.45) at post-test and 12-month follow-up. The results of these effect sizes are consistent with other studies on psychological treatments for other mental disorders, such as a meta-analysis by Baruch, Pistrang and Barker [34] on psychological interventions for bipolar disorder (Burden, g = -.80). Based on the results of these studies, we expect an effect size of 0.60 because the design of this study consists of two experimental groups. For the calculation of the sample size, we used the G*Power 3.1 software [35], taking into account an alpha of 0.05 and a statistical power of 0.80 in a two-tailed t-test. We need a sample size of 90 participants (45 relatives per condition) to reach an effect size of 0.60 for burden. Finally, we considered possible sample loss during the treatment program. Based on studies of interventions for relatives of people with borderline personality disorder, we expect a 30% dropout rate [17, 23, 27, 36, 37]. Therefore, taking this outcome into account, our study requires a total sample size of 124 participants (62 participants per experimental condition).

### Participants

Participants will be relatives of patients who meet criteria for SBD [38]. The diagnoses will be carried out by a specialist in psychology, and the sample will be recruited from different centers: the Clinical Hospital from the Valencia University and its Mental Health Services; the PREVI Clinical Center and its three centers in Castellon, Valencia, and Alicante; and associations of relatives of people with mental disorders from Valencia, Murcia, and Zaragoza, from November 2021 to May 2022.Thus it is an ongoing study.

In the case of the patients, the following inclusion and exclusion criteria will be established: 1) fulfill the diagnostic criteria for SBD; 2) be in the process of undergoing treatment or follow-up in one of the recruitment centers indicated; 3) give their agreement to participate in the study in writing by signing the informed consent form; in the case of minors, the consent form must be signed by their parents; 4) The presence of another serious pathology such as psychosis, schizophrenia, intellectual disability, etc. will be an exclusion criterion.

In the case of family members, the following inclusion criteria will be followed: 1) being a family member of one of the patients with a diagnosis of SBD who is undergoing treatment or follow-up in one of the recruitment centers indicated; 2) signing the informed consent. The presence of any pathology that keeps the intervention from being carried out (such as psychosis, schizophrenia, substance dependence, etc.) will be an exclusion criterion.

### Procedure

Family members of patients with SBD will be given the opportunity to participate in the study. Once they have completed the informed consent, an expert clinician will carry out the assessment of each participant to check that the inclusion and exclusion criteria are met, and an independent investigator, unaware of the characteristics of the study, will be contacted to carry out randomization. At all times, for randomization purposes, an experimenter from outside the research team will assign each family member to one of the two study conditions (FC-SBD or TAU-O) using a random number software program. The randomization sequence will be concealed from the clinicians participating in the study. Independent assessors will administer the assessment protocol to the participants without being aware of the experimental condition to which they belong (FC-SBD or TAU-O). Patients will also be assessed before starting the intervention programs for relatives. After the assessment has been completed, all the relatives will start to receive the intervention in the condition to which they have been assigned. In the case of patients, they will participate in their current treatment or care. At the end of the intervention, the assessment protocol will be readministered to both relatives and patients, again by independent assessors, and the same thing will be done at the six-month follow-up.

### Ethics

The project involves clinical experimentation with human participants. The investigators adhere to the Helsinki Convention and the Madrid Declaration of the World Psychiatric Association on clinical research. The present project has been submitted for approval to the Ethics and Research Committee of the University of Valencia, and when we receive approval from this committee (UV-INV_ETICA-1623849), the document will form part of the final documentation of the present study. All participants will be volunteers and will give their informed consent to participate in the study. All eligible participants will be given oral and written information about the study and the two intervention modalities. Specifically, they will be informed that they may leave the study at any time, without providing any explanation, and that this decision will in no way affect their family member's regular treatment at the center. Participant selection and evaluation will be carried out by qualified personnel who will not know the condition to which a given participant has been assigned. In the same way, the treatments will be carried out by qualified and expert professionals. Furthermore, it should be emphasized that the protocols for action and custody of the information followed at the University Clinical Hospital, and the PREVI Clinical Center, , where the participants will be recruited and the interventions will be carried out, comply with all the requirements of the LOPD (Organic Law 15/1999, of 13 December 2018). In addition, according to the existing knowledge in this field so far, we have not found any risks for the participants; however, the appearance of any important clinical change that would involve any type of risk would not only imply removal of the participant from the project, but also his/her referral for specialized study and care. The assessment protocol described above is composed of standardized instruments that are risk-free for the participants (interviews, questionnaires, registers). The intervention protocols are based on empirically validated treatments (cognitive-behavioral oriented) designed and developed by staff with extensive knowledge and experience in this field. For ethical reasons, participants in the control condition, where the psychoeducation protocol (TAU-O) will be applied, will be given the opportunity to receive the FC-SDB program if they so desire, even if the study has already ended. The study was registered at clinicaltrials.gov as ID: NCT05157607

### Interventions

#### Interventions with family members

##### Family Connections Protocol for Relatives of Patients with SBD (FC-SBD)

The intervention lasts three months and includes 12 sessions with a weekly two-hour group format.

The FC program [23] is divided into six modules:

- Module 1: Up-to-date information and research on suicide (Epidemiology, frequency, Risk factors, protective factors).
- Module 2: Psychoeducation on the development of suicide, explanatory theories, available treatments, comorbidity.
- Module 3: Emotional regulation skills, skills of acceptance, validation, approach, awareness, and to decrease emotional reactivity.
- Module 4: Skills to improve the quality of relationships in family interactions (letting go of guilt and anger, acceptance skills in relationships).
- Module 5: Communication skills and effective self-expression.
- Module 6: Problem management and making safe plans for crisis management.
- All the modules include practice exercises, video viewing, and homework assignments. In addition, throughout the program, with the aim of increasing social support, the FC-SBD program provides a forum where participants can stay in touch and share common problems and solutions.

##### Treatment as Usual Optimized Protocol (TAU-O)

Family members in this condition will continue to receive their treatment as usual in their care center of reference. In addition, we will optimize the treatment based on the recommendations of the international guidelines for the treatment of suicide. There will be one three-hour session in group format with the following component:

- Module 1: Updated information and research on suicide (Epidemiology, frequency, Risk factors, protective factors). Psychoeducation on the development of Suicide, Explanatory theories. Available treatments, and comorbidity.

In both conditions, after each face-to-face session, the participant will be asked to review the contents covered during the session as homework (independently of the homework corresponding to the specific module being addressed). All interventions carried out with family members will be performed by clinical psychologists or general health psychologists with at least a Master's degree or a doctoral degree and previous training in the administration of the programs.

### Treatment of patients

With regard to the treatment of patients, the routine treatment they receive in their centers of reference will be followed. All interventions will be carried out by the clinical psychologists and psychiatrists working in these centers.

### Measures

After reviewing the literature, an evaluation protocol was designed that includes the main instruments that have been commonly used by authors working in this field [17, 23, 39], as well as some additional instruments that we consider relevant to the hypotheses proposed. In all cases, the Spanish validations of the assessment instruments will be used; if they do not exist, the validations will be carried out by the researchers of this study.

### Measures - Relatives (participants)

**Data on demographic variables**: age, sex, educational level, income, marital status, number/age of children, and history of psychological treatment.

- 1. Measures of primary outcomes

#### Burden assessment scale [40]

This 19-item scale assesses two dimensions of caregiver burden of a loved one's illness (objective and subjective) in the past six months. The items are rated on a 4-point Likert scale (1-4), where higher scores indicate higher levels of illness burden. The psychometric properties of this scale are adequate, with an internal reliability between .89 and .91 and adequate validity [41].

- 2. Measures of secondary outcomes

##### Register of critical incidents with the family member with SBD

This register was developed ad hoc for this study. The questions recorded are the following: frequency of suicide attempts in the past six months, number of days of self-harm in the past six months, number of episodes of verbal/physical violence with caregivers in the past six months; frequency of visits to the psychiatric emergency room in the past six months, frequency of therapy sessions conducted out of schedule in the past six months (face-to-face, phone calls, etc.).

##### Family empowerment scale [42]

This scale has a total of 34 items. It is composed of three subscales referring to attitudes, knowledge, and behaviors related to (1) Family, (2) the Service System, and (3) Community Participation. The items are rated on a Likert scale (1-5), where higher scores show greater feelings of empowerment. Both the validity and reliability of this scale are adequate, and the internal consistency of the subscales shows coefficients between α = .87 and α =.88.

##### Depression, anxiety, and stress scale [43, 44]

We have used the short, validated Spanish version with 21 items on the frequency of negative emotional symptoms in the past week. The items are rated on a Likert scale (0-3) where the higher the score, the higher the frequency of symptoms of depression, anxiety, and/or stress. The internal consistency of the scale was excellent, with Cronbach's alphas for the DASS-21 subscales: Depression (α = .94), Anxiety (α = .87) and Stress (α = .91) [45].

##### Difficulties in emotion regulation scale - Spanish version [46]

We used the Spanish validation containing 28 items. This questionnaire is divided into five subscales: (1) Lack of emotional control, (2) Life interference, (3) Emotional inattention, (4) Emotional confusion, and (5) Emotional rejection. The items are rated on a Likert scale (1-5) where higher scores indicate greater difficulty in regulating emotions. Psychometric properties are excellent, with an internal consistency of α = .93 and test-retest reliability of pl = .74, p < .001.

##### Quality of life index-Spanish version [47]

It consists of a 10-item index of perceived quality of life. It refers to physical and emotional well-being, functioning at work, satisfaction with personal relationships and self-independence, support in the community and from an emotional point of view, spiritual well-being, and perceived overall quality of life. The items are rated on a Likert scale (0-10) where higher scores indicate higher perceived quality of life. The psychometric properties are good for both internal consistency (α = .89) and test-retest reliability (r = 0.87).

##### Opinion and Expectations of Treatment Scale

This scale was designed and developed by members of the research team and constructed from an adaptation of another opinion and expectations questionnaire [48]. The constructs this scale assesses are: opinion, acceptance and satisfaction with the skills training program, and the changes in the participants after the completion of each module. The questions refer to the rationale for the intervention, recommendation of the program, satisfaction with the program, usefulness and expectations of the skills training. The items are rated on a Likert-type scale ranging from 0 "Not at all" to 10 "Very much".

##### Qualitative Assessment

After the intervention ends, a focus group will be carried out with 25 participants in order to record family members’ personal opinions about the intervention. A focus group is a qualitative research method that provides information about a specific topic from the perspective of a group of people who share a central element of their experience, reducing the influence of the researcher during the process. In this case, participants will be asked an open question about their willingness to repeat or recommend FC-SBD, and a psychologist will write down each participant's answer.

### Measures - Patients

**Data about demographic variables:** age, sex, educational level, income, employment status, marital status, number/age of children, and history of psychological treatment.

1. Measures of primary outcomes

#### Register of critical incidents with the family member with SBD.

2. Measures of secondary outcomes

##### Interpersonal Needs Questionnaire [49, 50]

We use the Spanish version of this 15-item questionnaire that assesses the degree of dissatisfaction with their need to belong (frustrated belonging) and the degree to which they perceive themselves as a burden to others (perceived burden). The items are rated on a Likert-type scale (1-7) where higher scores indicate higher levels of frustrated belonging and perceived burden to others. Psychometric properties were good: scale reliability was very good (perceived burden, α = 0.96; and frustrated belonging, α = 0.78).

##### Patient Health Questionnaire [51, 52]

It consists of a nine-item questionnaire that assesses depressive symptoms in the past two weeks. The items are rated on a Likert scale (0-3) where higher scores indicate higher frequency of depressive symptoms. The severity of depression on this questionnaire is measured through the total score, which can be categorized as none or minimal, mild, moderate, moderately severe, and severe. Validity has been adequate, with a sensitivity of 88% and a specificity of 88% for major depression.

##### Overall Anxiety Severity and Impairment Scale [53, 54]

We use the Spanish version of this questionnaire, which consists of a five-item instrument that assesses the frequency and intensity of anxiety symptoms in the past week. In addition, it measures interference in work and academic, social, and daily life domains, as well as avoidance behaviors. The items are rated on a Likert-type scale (0-4). The psychometric properties are good in terms of internal consistency (α= 0.86), convergent and discriminant validity, and sensitivity to change (α= 0.86).

##### Validating and Invalidating Responses Scale [55]

It consists of a 16-item scale on the validation and invalidation of family members' responses about their loved ones. It is divided into two subscales (validation and invalidation), and the items are rated on a Likert scale (0-4) where higher scores indicate higher perceived validation or higher perceived invalidation (depending on the subscale).

##### Lum Emotional Availability of Parents [56]

This 15-item questionnaire measures the perceived emotional availability of primary caregivers. The items are rated on a Likert scale (1-6) where higher scores indicate greater emotional availability of caregivers. Psychometric properties were excellent for both subscales (mother, α = .9; and father, α = .93). In addition, test-retest reliability was also adequate for the mother's subscale (r = .92) and the father's subscale (r = .85).

### Data analyses

Regarding data analysis, the CONSORT guidelines [57] will be followed. First, participants' scores in the two conditions before receiving the intervention will be compared to check that there are no significant differences between them on the outcome measures and that they are, therefore, comparable after randomization. ANOVAs will be conducted for continuous variables and Chi-square tests for categorical variables. For outcome measures at post-treatment, we will study whether the assumption of homoscedasticity is met with Leven's test. If this assumption is met, repeated-measures MANOVAs and F-tests will be used to compare the two experimental conditions. If the homoscedasticity assumption is not met, the Brown-Forsythe test will be applied. F-tests for statistical significance will be followed by post hoc comparisons. In particular, Tukey will be used when the homoscedasticity assumption is met, and Games-Howell if the homoscedasticity assumption is not met. Appropriate analyses will also be carried out to calculate intervention effect sizes and confidence intervals. The intention-to-treat principle will be used when analyzing pre- and post-treatment data and at six-month follow-up, using mixed-effects models with full information and maximum likelihood estimation. This method has been recommended due to its flexibility in handling missing data [58]. To complement the MANOVA results and post hoc comparisons, effect sizes will be calculated using the standardized mean difference proposed by Cohen [59]. These effect sizes will be calculated to assess changes within and between groups, all based on a pooled standard deviation. Although per-protocol analyses (only analyses of data from participants who complete treatment) suffer from selection bias, they will also be conducted because they allow conclusions to be drawn about the maximum efficacy of the intervention in participants who are fully compliant with treatment. However, when the trial is over, the analytical methodology for controlled clinical trials will be reviewed before analyzing the data to select the most appropriate analytical procedures.

For the qualitative study, semi-structured, in-depth interviews will be used. These interviews will follow the guidelines of Knox and Burkard [60]. The research design will be carried out following the criteria established by Cooke, Smith and Booth [61]. Data will be analyzed using the consensual qualitative research (CQR) method. This methodology has been developed by clinical psychologists to specifically investigate the experience of individuals undergoing psychological treatment [62]. Data reporting will be carried out following the COREQ guidelines [63]. Furthermore, we will attempt to meet the criteria for quality in qualitative research recently developed by Levitt et al. [64] in order to pursue what the authors refer to as methodological integrity in the qualitative field.

## Discussion

Family members of people with SBD are disoriented, guilty, hopeless, and have a strong feeling of being burned out [14], and they are routinely excluded from intervention programs for patients with SBD. For this reason, it is necessary to develop interventions designed to teach them emotional regulation and coping skills that allow them to cope with their relationship with the patient with SBD [19]. FC is a program that has been shown to be effective in reducing burden, depression, and anxiety, and in increasing dominance and validating behaviors in relatives of people with BPD [17, 23, 25–29]. However, there are no RCTs that demonstrate the efficacy of this program in patients with SBD. For this study, we have adapted the original FC [23] to relatives of people with SBD (FC-SBD).

The first objective of this study is to analyze the efficacy of an adaptation of FC for relatives of people with SBD. For this purpose, we have designed an RCT that compares an experimental condition (FC-SBD) and a control condition, which will be an active condition and will also be optimized with a psychoeducational session on SBD (TAU-O). The sample comes from different centers specialized in the treatment of people with SBD, as well as from associations of relatives of people with mental disorders.

The second objective is to analyze the acceptability of this program by the participants. In addition to analyzing the change in the participants, it is important to analyze the extent to which they accept and are comfortable with the program and their opinions and expectations about it, in order to improve adherence and future implementation.

To our knowledge, none of the previous studies about FC evaluated whether the change produced in family members was associated with an improvement in the emotional state of the diagnosed patients (e. g [17].). For this reason, another objective is to analyze whether the possible reduction in emotional load, depression, and anxiety in family members after the intervention with FC-SBD is associated with an improvement in depression, anxiety, and perception of the family environment in patients with SBD. The fourth objective is to analyze whether the improvements in emotional regulation, validation, control and empowerment, and family functioning predict the reduction in burden, anxiety, and depression and the improvement in the quality of life of relatives. Finally, we will analyze the opinions, preferences, and perceptions of the relatives in both treatment conditions.

Previous versions of FC focused exclusively on relatives of people with BPD. We want to emphasize that, to date, this is the first RCT carried out to examine the effectiveness of the FC program in participants with SBD. This allows relatives of different patients with very different diagnoses, such as personality disorder, depression disorder, eating disorders, etc., to benefit from this intervention. Finding treatment programs that are effective in reducing burden, hopelessness, and depression and improving the family climate in relatives of people with SBD could allow us to improve suicide prevention in the family environment.

In addition, this study will be carried out with a Spanish-speaking sample of Spanish relatives, which will allow us to demonstrate the efficacy in this population. Most of the published studies on the efficacy of FC have been conducted in English-speaking samples (e.g. [26–29]), and so our results can be compared with those obtained in English-speaking countries.

Furthermore, we compare the FC-SBD in an RCT with an active group (TAU-O). Family members in the TAU-O condition are undergoing treatment in their usual center, and we also add a psychoeducational session on suicide. Thus if our hypotheses are supported, we can demonstrate the effectiveness of FC-SBD, which will accelerate the implementation of this program in clinical centers.

Our study has some limitations. The main limitation is that, although one objective is to evaluate the effect of FC on patients, access to them is very difficult because patients may not be in contact with family members, and sometimes there is no relationship between them. For this reason, they are difficult to access. Another limitation is that the follow-up is at six months, and it would be advisable to do a follow-up at 12 months to check the evolution of burden, depression, anxiety, and other clinical variables of the relatives after participating in FC-SBD.

Suicide attempts are great challenges faced by families, and they produce significant feelings of guilt, fear, and hopelessness [14–16]. Therefore, it is necessary to conduct studies that help family members to improve their relationships with patients with SBD. We hope that this study will provide results that confirm the efficacy of the FC program in relatives of people with SBD. These results will also confirm its good acceptance by family members and help us to find out whether it is a good program to improve the prevention of suicidal behaviors in the family environment.

## Data Availability

The data that support the findings of this study are available from Jose H. Marco but restrictions apply to the availability of these data, which were used under license for the current study, and so are not publicly available. Data are however available from the authors upon reasonable request and with permission of Jose H. Marco.

## Abbreviations

SBD: Suicidal Behavior Disorder
FC: Family Connections
FC-SBD: Family Connections for Suicidal Behaviour Disorder
CONSORT: Consolidated Standards of Reporting Trials
SPIRIT: Standard Protocol Items: Recommendations for Interventional Trials
TAU-O: Treatment As Usual Optimized
RCT: Randomized Controlled Trial
MANOVA: Multivariate analysis of variance
ANOVA: Analysis of variance
